# Use of a Cumulative Exposure Index to Estimate the Impact of Tap Water Lead Concentration on Blood Lead Levels in 1- to 5-Year-Old Children (Montréal, Canada)

**DOI:** 10.1289/ehp.1409144

**Published:** 2015-06-16

**Authors:** Gerard Ngueta, Belkacem Abdous, Robert Tardif, Julie St-Laurent, Patrick Levallois

**Affiliations:** 1 Axe Santé des populations et pratiques optimales en Santé, Centre de recherche du CHU (Centre hospitalier universitaire) de Québec, Québec, Québec, Canada; 2 Département de médecine sociale et préventive, Faculté de médecine, Université Laval, Québec, Québec, Canada; 3 Département de santé environnementale et de santé au travail, École de Santé Publique de l’Université de Montréal (ESPUM), Montréal, Québec, Canada; 4 Direction de la santé environnementale et de la toxicologie, Institut national de santé publique du Québec, Québec, Québec, Canada

## Abstract

**Background:**

Drinking water is recognized as a source of lead (Pb) exposure. However, questions remain about the impact of chronic exposure to lead-contaminated water on internal dose.

**Objective:**

Our goal was to estimate the relation between a cumulative water Pb exposure index (CWLEI) and blood Pb levels (BPb) in children 1–5 years of ages.

**Methods:**

Between 10 September 2009 and 27 March 2010, individual characteristics and water consumption data were obtained from 298 children. Venous blood samples were collected (one per child) and a total of five 1-L samples of water per home were drawn from the kitchen tap. A second round of water collection was performed between 22 June 2011 and 6 September 2011 on a subsample of houses. Pb analyses used inductively coupled plasma mass spectroscopy. Multiple linear regressions were used to estimate the association between CWLEI and BPb.

**Results:**

Each 1-unit increase in CWLEI multiplies the expected value of BPb by 1.10 (95% CI: 1.06, 1.15) after adjustment for confounders. Mean BPb was significantly higher in children in the upper third and fourth quartiles of CWLEI (0.7–1.9 and ≥ 1.9 μg/kg of body weight) compared with the first (< 0.2 μg/kg) after adjusting for confounders (19%; 95% CI: 0, 42% and 39%; 95% CI: 15, 67%, respectively). The trends analysis yielded a *p*-value < 0.0001 after adjusting for confounders suggesting a dose–response relationship between percentiles of CWLEI and BPb.

**Conclusions:**

In children 1–5 years of age, BPb was significantly associated with water lead concentration with an increase starting at a cumulative lead exposure of ≥ 0.7 μg Pb/kg of body weight. In this age group, an increase of 1 μg/L in water lead would result in an increase of 35% of BPb after 150 days of exposure.

**Citation:**

Ngueta G, Abdous B, Tardif R, St-Laurent J, Levallois P. 2016. Use of a cumulative exposure index to estimate the impact of tap water lead concentration on blood lead levels in 1- to 5-year-old children (Montreal, Canada). Environ Health Perspect 124:388–395; http://dx.doi.org/10.1289/ehp.1409144

## Introduction

Adverse effects of lead in children have been widely studied for years. Exposure to lead has been linked to anemia (Flanagan et al. 1982; Jain et al. 2005; Schwartz et al. 1990; Waldron 1966), renal dysfunction (de Burbure et al. 2006), impaired hearing and postnatal growth [U.S. National Toxicology Program (NTP) 2012], and neurotoxic effects (Lidsky and Schneider 2003; Ronchetti et al. 2006). Pooled results of cohort studies indicated that, in young children, blood lead levels (BPb) < 7.5 μg/dL were associated with intellectual deficit, without any obvious threshold (Lanphear et al. 2005).

Drinking water has been identified as a source of oral exposure to lead (Brown et al. 2011; Triantafyllidou and Edwards 2012). Children can absorb 40–50% of an oral dose of water-soluble lead, compared with 3–10% for adults [Agency for Toxic Substances and Disease (ATSDR) 2007]. Despite great interest for the influence of environmental lead on children’s health, few studies have investigated the impact of water lead levels (WLL) on BPb in those < 6 years of age. However, young children represent the most sensitive population and are at higher risk of deleterious effects of lead from drinking water (ATSDR 2007; Gulson et al. 1997; Triantafyllidou and Edwards 2012). A few epidemiologic studies including children < 6 years of age have reported a strong association between WLL and BPb (Lacey et al. 1985; Lanphear et al. 1998, 2002; Levallois et al. 2014), but others have not (Gasana et al. 2006; Rabinowitz et al. 1985).

The distribution of absorbed lead to organ systems appears to be quite similar in children and adults (ATSDR 2007; Barry 1975; Gross et al. 1975), with bone lead accounting for about 73% of the body burden in children (ATSDR 2007; Barry 1975). It has been reported that the half-life of blood lead is approximately 30 days for adults [ATSDR 2007; World Health Organization (WHO) 1995]. For young children, to our knowledge, no specific value has been reported. Duggan (1983) considered that the clearance rate of blood lead may be higher in children than in adults. However, the true value of this half-life, though shorter, might be very speculative. The time required to reach the steady-state is about five to six times the elimination half-life time (i.e., 5–6 months) after a repeated exposure (Greenblatt 1985). Therefore, BPb at a given time is related to the cumulative exposure over the previous 5 months. That is, 50% of blood lead is eliminated after approximately 1 month, 75% after 2 months, 87.5% after 3 months, etc.

Several authors reported that lead concentration in tap water increases with water temperature (Cartier et al. 2011; Schock 1990), suggesting that WLL is likely to show seasonal fluctuations. More recently, we reported marked winter-to-summer changes in WLL in the Montreal area and a potential impact on children’s BPb using the integrated exposure uptake biokinetic model (IEUBK) (Ngueta et al. 2014). The geometric means of WLL (± SE) were 2.7 ± 2.2 μg/L during winter and 8.1 ± 1.5 μg/L during summer. However, previous cross-sectional studies did not take into account the fact that the tap water lead concentration may vary seasonally and did not consider the cumulative exposure to lead from water in the months preceding the blood sampling (Gasana et al. 2006; Lanphear et al. 1998; Levallois et al. 2014; Morse et al. 1979; Oulhote et al. 2013). Although the IEUBK model integrates several biological parameters, it assumes that exposure levels are stable over the year (Mickle 1998), and it does not capture cumulative lead exposure over time.

In the present study, we addressed the gap in knowledge about time-dependent cumulative lead exposure from tap water in relation with children’s BPb. More specifically, we aimed to estimate the dose–response relationship between drinking-water cumulative lead exposure and BPb in 1- to 5-year-old children. Given the low clearance of blood lead, a more accurate way to estimate cumulative exposure to lead should substantially improve our ability to accurately estimate the effects of lead exposure resulting from water.

## Methods

*Population study*. Children 1–5 years of age were recruited from four neighborhoods of Montreal (Quebec, Canada) selected for the possible presence of lead pipes and old houses. Details of the recruitment process were described previously (Levallois et al. 2014), and eligibility criteria are depicted in the Supplemental Material, Figure S1. A randomly selected list of 9,500 families, with at least one child 1–5 years of age and living in the targeted boroughs, was obtained from the Quebec government’s health database (Régie de l’assurance maladie du Québec). Only one child per family was randomly selected. After excluding families living in buildings with more than three dwellings, an information letter with a consent form was sent to 3,800 families living in the targeted boroughs. Of the 3,800 families contacted by letter, 2,661 were reached by phone to verify their eligibility. A total of 567 of them refused to participate before assessing eligibility (21.3%). Of the 549 eligible families identified, 214 additional guardians declined to participate, leading to a total proportion of 29.3% of refusal rate among families assessed for admissibility. Finally, 57% of eligible families (*n* = 313) were included in the study. Informed consent was obtained from the guardians of each child. The data collection procedure was approved by the ethics committees of the CHU (Centre hospitalier universitaire) de Québec and Health Canada. The present study was also approved by the Research Ethics Board for Health Sciences of Université Laval (Canada).

*Data collection*. The main survey involving 313 homes was undertaken between 10 September 2009 and 27 March 2010. During this campaign, blood and environmental samples (water, dust, and paint samples) were collected together with information about participating children and their guardians’ characteristics (Levallois et al. 2014). Briefly, a questionnaire was administered by a trained nurse to collect information on child’s characteristics and lifestyle habits, parents’ characteristics and lifestyle habits, child’s nutrition (e.g., daily water consumption patterns, frequency of meals), and the total number of persons living in each household. Another questionnaire was administered by a hygienist technician to parents to collect information about home environment and characteristics (e.g., nearby industry, distance to roadway).

Throughout the main survey, the water temperature was measured after 3 min of flushing; it varied between 1.4°C and 21.7°C (ambient temperature, –15.3°C to 23.8°C). The main survey did not cover the summer period. Given that previous studies suggested the influence of water temperature on lead concentration in tap water (Cartier et al. 2011; Karalekas et al. 1983), a second survey took place from 22 June 2011 to 6 September 2011 and was limited to collecting water samples in 100 households randomly drawn from the initial sample households visited during the first survey. These included 80 homes with lead service lines and 20 without lead service lines. During this second campaign, the water temperature after 3 min of flushing varied between 16.0°C and 24.1°C (ambient temperature, 20–28°C).

Water sampling. During each home visit, five samples of tap water were collected from the kitchen by the environmental technician without removing the tap aerator. Water samples were collected in pre-acidified plastic containers and kept at approximately 4°C until the laboratory analysis. The first 1-L sample was taken after 5 min of flushing (5MF), at usual flow (7–12 L/min). Thereafter, a stagnation time of 30 min was observed without any use of water in the household. Subsequently, four consecutive 1-L samples were collected from the first water draw (30MS1, 30MS2, 30MS3, and 30MS4, respectively). The same procedure was used for both campaigns.

Dust and paint sampling. Floor dust was sampled with wet wipe in the center of the available floor space in three different rooms of the home of each participant: the child’s room, home entrance, and another room frequently used by the child. Windowsill dust was also sampled in the child’s room. The lead content of the interior painted surfaces of homes was evaluated with an X-ray fluorescence (XRF) analyzer (Niton XL3t-300; Elemental Controls, Mississauga, Ontario, Canada). When there was chipped paint, at least 200 mg for all paint-chip samples from the home were collected for laboratory analyses. The details of this evaluation were reported previously (Levallois et al. 2014).

Blood collection. One sample of venous blood (between 2 and 4 mL) was drawn from the child’s arm by a trained nurse and kept in a Becton-Dickinson tube (BD-367863) pretreated with anticoagulant EDTA (Ethylenediaminetetraacetic acid) at 4°C until the laboratory analysis.

*Laboratory analyses*. Lead analyses in water, dust, paint, and blood samples were described in details previously (Levallois et al. 2014; Ngueta et al. 2014). In brief, water samples analyses from the first survey were performed by an accredited ISO-17025–certified laboratory according to U.S. Environmental Protection Agency (EPA) protocol (U.S. EPA 1994). For the second campaign, lead analyses were performed by the Centre d’expertise en analyse environnementale du Québec (Provincial reference laboratory). The protocol used was very similar to the previously described U.S. EPA protocol. However, the U.S. EPA protocol was modified with a 24-hr digestion time instead of 16 hr to retrieve the particle portion of lead. Each sample was analyzed by inductively coupled plasma mass spectroscopy (ICP-MS) using selection ions mode. For water analyses, quality control was regularly performed during the analysis period (blank, certified reference material, duplicate, and fortified blank). The blank sample (i.e., a sample of ultra-clean water) was introduced for every 20 samples. The lead concentration in blank samples, if present, was below the detection limit. The correlation coefficient for duplicates was 0.99. Results obtained for fortified blanks were within the limits used by the laboratory. The detection limit for the method was 0.01 μg/L and the quantification limit was 0.02 μg/L.

For dust analyses, each wet wipe was placed individually in plastic tubes and kept at 4°C until laboratory analysis. Two different wipe controls were used for each sampling zone: *a*) A control wet wipe (in one of every two residences) was manipulated outside the plastic tube but without wiping on a surface, and *b*) a control template was held in the air according to the regular protocol (once a week), and the wipe was used to make the S-like motion inside the template. Analyses of the lead dust wipes were performed according to a standardized method (ASTM E-1728-03) and consisted of predigesting the wet wipe in a partially covered 50-mL tube with 2 mL of concentrated nitric acid at room temperature for 5 hr. The digestion tube was then placed in a bath at 80°C for 12 hr. Afterward, the tube was withdrawn from the bath, and when it reached room temperature, 1 mL hydrochloric acid was added and a total volume of 10 mL was achieved by adding deionized water.

Paint chips were collected when present on damaged wall surfaces or flooring for laboratory analyses. Samples were digested at room temperature for 2 hr in a partially covered test tube containing 2 mL of concentrated nitric acid. Afterward, samples were covered and placed in an oven at 110°C for 18 hr.

For both dust and paints, total lead analyses were performed using ICP-MS (Elan-6000; PerkinElmer). Certified standard reference material paint chips from the National Institute of Standards and Technology (NIST 1579A) and demineralized water reference material from Ultra Scientific (ICM 240) were used for calibration and quality control. For paints, the detection limit was 10 μg/g and the quantification limit was 30 μg/g. For dust samples, the detection limit was 0.01 μg and the quantification limit 0.015 μg per sample.

Whole blood samples were analyzed for lead content by ICP-MS at the laboratory of the Institut national de santé publique du Québec (INSPQ, Québec, Canada). Quality controls and detection and quantification limits were previously reported (Levallois et al. 2014). The detection limit was 0.02 μg/dL and the quantification limit 0.08 μg/dL. Internal quality control was conducted using three reference materials obtained from the INSPQ (External Quality Assessment Schemes; 1.87 μg/dL, 6.25 μg/dL, and 30 μg/dL). Duplicates performed every 10 analyses had a correlation coefficient of 0.99.

*Estimation of cumulative water lead exposure index*. In the first step, we modeled the seasonal changes in mean WLL, as observed in Montreal (Canada) in 2009–2010, to obtain the estimated values of daily lead concentrations in tap water. This modeling was reported in detail elsewhere (Ngueta et al. 2014). Briefly, we modeled the average temporal (daily) changes in WLL for the population as a whole using a nonlinear regression model (WLL = A × Sinus[(2π/365.2) × (Days-B)] + C). The coefficients A, B, and C and predicted WLL for each day of the year were estimated after adjusting for the presence of lead service lines (yes/no), flow rate (continuous), neighborhood (four categories), type of residence (single family home vs. multiple family), age of dwelling (continuous), total number of people living in the household, and floor where the tap was located (ordinal). The nonlinear regression modeling then generated the predicted WLL for each day of the year. For each of the involved households, we calculated the arithmetic mean of WLL from the five 1-L water samples. The average value reflected the exposure level if the child consumes 20% of flushed and 80% of stagnant sample (namely, “80:20” scenario). The nonlinear regression model as described above was performed for the average value.

Assuming the elimination half-life time of blood lead of 30 days, and based on the daily amount of ingested water as reported in the in-home personal interview questionnaire, we retrospectively cumulated the estimated values of the daily lead uptake over 150 days (≈ 5 months), taking into account the daily elimination rate and assuming that the transfer of lead from blood to tissues follows a first-order kinetics (Leggett 1993). The amount of lead that still remains in blood at time *t* was defined as B_t_.

B*_t_* = B_0_ × e^–(Ln 2)/30 ×^ *^t^*,

where B_0_ represents the initial quantity of lead in the blood.

For each child, if the mean WLL expected at the day *i* is defined as WLL*_i_* (in micrograms per deciliter), then the amount of ingested lead at that day would be WLL*_i_* × Q*_i_*, where Q*_i_* represents the amount of water ingested at day *i* (in liters). The amount of ingested lead reaching the bloodstream is expected to be WLL*_i_* × Q*_i_* × *k*, where *k* represents the gastrointestinal absorption rate of lead from the media considered. Based on the prior works, this value is approximately 0.50 for water (U.S. EPA 2002; White et al. 1998; Yu et al. 2006). At the day *i + 1*, the amount of lead that remains in blood is theoretically as follows:

B*_i_*_+_ *_1_* = (WLL*_i_* × Q*_i_* × *k*) × e^–(Ln 2)/30 × (^*^1^*^)^ + (WLL*_i + 1_* × Q*_i + 1_* × *k*).

For the day *i + 2*, the corresponding expected value is

(WLL*_i_* × Q*_i_* × *k*) × e^–(Ln 2)/30 × (^*^2^*^)^ + (WLL*_i + 1_* × Q*_i + 1_* × *k*) × e^–(Ln 2)/30 × (^*^1^*^)^ + (WLL*_i + 2_* × Q*_i + 2_* × *k*).

Given that uptake and elimination are daily processes, the CWLEI was developed by following the same scheme, and the exposure was then retrospectively cumulated over the 5 months (150 days) preceding the day of blood collection (*i* from 0 to 150).

The cumulative lead exposure index through drinking water (CWLEI) was then estimated as follows:

*CWLEI* = Q_e_ × 0.50 × ^^150^^Σ*__i__*__= 0__[*WLL_i_* × e^–(Ln 2)/30 × (^*^n – i^*^)^] (expressed in micrograms),

where *n* represents the number of days considered for cumulating exposure (*n* = 150). The daily amount of water intake was considered as constant, namely Q_e_.

*Statistical analysis*. The outcome variable in the present study was the child’s BPb (micrograms per deciliter). The exposure variable of interest was CWLEI divided by body weight on the day of the first home visit. We used causal diagram to decide which variables to control for in our statistical analysis (see Supplemental Material, Figure S2). For this, we used the DAGitty software (Textor et al. 2011) and the algorithm developed for this browser-based environment, as described in detail elsewhere (Textor and Liskiewicz 2011). The minimal sufficient adjustment sets identified for estimating the association between CWLEI and BPb included child’s age (categorized into quintiles), child’s sex, child’s ethnicity (Caucasian, other), duration of breastfeeding (in months), mother’s education level (< secondary, secondary, postsecondary), frequency of child care attendance (days per week), number of meals per day (≤ 2, > 2), and the season of blood collection (autumn or winter). We finally adjusted for these variables in the first modeling. We also performed a second set of models that were additionally adjusted for lead in paint (XRF < 1 mg/cm^2^, XRF ≥ 1 mg/cm^2^ or paint chips < 5,000 mg/kg, ≥ 5,000 mg/kg), floor dust lead loading (micrograms per square feet), and windowsill dust loading (micrograms per square feet), with dust and windowsill lead loadings modeled as categorical variables in quintiles.

Main analyses. SAS software (version 9.3; SAS Institute Inc., Cary, NC) was used for all analyses. The UNIVARIATE procedure was used to assess distribution of continuous variables. The FREQ procedure was used to describe categorical variables. The Student *t*-test and ANOVA (analysis of variance) procedure were used to compare BPb across strata of a given covariate. For checking the presence of multicollinearity, we referred to condition number as well as proportion of variances with respect to each independent variable (Schroeder 1990).

Because the outcome variable (BPb) was skewed, we used a natural logarithmic (ln)transformation to normalize the distribution before analyses. We performed the REG procedure to estimate the association between CWLEI and ln(BPb). The CWLEI was modeled as a continuous variable and then categorized into quartiles. Estimates from the categorical model were exponentiated to derive the ratio of the geometric mean for each quartile relative to the geometric mean of the lowest quartile. For estimating the trend *p*-values, we used the geometric mean for each quartile to code the exposure variable, and the latter was then introduced into the regression model as an ordinal variable. All *p*-values reported were two-sided, and the statistical significance was assumed for a *p*-value < 0.05.

Sensitivity analyses. The development of the CWLEI relies on several assumptions. We considered the 50% gastrointestinal absorption rate; and for modeling, we assumed that children consumed on a daily basis 80% of stagnant water and 20% of flushed water. To test the robustness of our index of exposure, we conducted a sensitivity analysis to assess the change in the CWLEI (and its association with BPb) with gastrointestinal rate. Although the value of 50% is commonly used in most previous works, O’Flaherty (1993) estimated that this value is a minimum. We then considered the scenarios where the child absorbed 50%, 75%, and 90% of ingested lead, respectively. We further considered for each of these scenarios the case where children consumed stagnant water, exclusively (100:0), 80% of stagnant water and 20% of flushed water (80:20), 50% of stagnant water and 50% of flushed water (50:50), 20% of stagnant water and 80% of flushed water (20:80), and fully flushed water, exclusively (0:100).

## Results

From the 313 children meeting the inclusion criteria, 8 were excluded because of missing blood values (*n* = 7) or the absence from home for an entire month before the home visit (*n* = 1). Seven additional children were excluded because their home remained unclassifiable with regard to the presence/absence of lead service lines and/or we missed data required for estimating the CWLEI (e.g., the daily amount of water intake). Of the 298 children included in the present analysis, 49 (16.4%) were < 24 months old, 65 (21.8%) were 24–35 months old, 91 (30.2%) were 36–47 months old, and 94 (31.6%) were 48–72 months old (Table 1). Girls represented 50% of children, and about 67% were Caucasians. Approximately 62% of mothers declared they had a university diploma, and 60% of guardians were owner of their residence. Blood samples were collected during winter for about 64% of children. The average daily water intake was 0.25 L in children ages 12–23 months, 0.29 L in children ages 24–35 months, and 0.35 L for those ages 36–72 months. As a whole, the mean BPb was 1.34 μg/dL [95% confidence interval (CI): 0.50, 3.61], and only 5 of included children had BPb exceeding the current standard of U.S. Centers for Disease Control and Prevention (CDC) (i.e., 5 μg/dL) (CDC 2012). Results from bivariate analyses with geometric mean (GM) of BPb across different characteristics strata are shown in Table 1. BPb was significantly higher in relation with non-Caucasian ethnicity, unemployed mother, mother with less than secondary degree, frequency of home cleaning ≥ 1/week, child care attendance, and autumn season.

**Table 1:** Blood lead levels by sociodemographic variables, guardians’ characteristics, season of blood collection, and environmental covariates.

Variable	*n* (%)	Blood lead levels (μg/dL)	
		GM (95% CI)	*p*‑Value^*a*^
Total	298 (100)	1.34 (0.50, 3.61)
Season of blood collection
Autumn	107 (35.9)	1.50 (0.57, 3.98)	0.002
Winter	191 (64.1)	1.24 (0.46, 3.32)
Child-related variables
Sex
Male	149 (50.0)	1.31 (0.48, 3.53)	0.431
Female	149 (50.0)	1.37 (0.51, 3.70)
Age (months)
12–24	49 (16.4)	1.32 (0.49, 3.55)	0.818
24–36	65 (21.8)	1.41 (0.45, 4.46)
36–48	90 (30.2)	1.31 (0.47, 3.65)
48–72	94 (31.6)	1.33 (0.57, 3.10)
Ethnicity
Caucasians	199 (66.8)	1.25 (0.50, 3.11)	0.003
Non-Caucasians	99 (33.2)	1.53 (0.51, 4.61)
Duration of breastfeeding (months)
< 6	51 (17.1)	1.49 (0.52, 4.32)	0.033
6–8	46 (15.4)	1.15 (0.50, 2.63)
8–10	42 (14.0)	1.16 (0.46, 2.90)
10–15	60 (20.1)	1.36 (0.51, 3.67)
≥ 15	59 (19.7)	1.43 (0.47, 4.36)
Missing values	41 (13.7)	1.41 (0.58, 3.41)
Number of meals/day
≤ 2	25 (8.4)	1.55 (0.59, 4.05)	0.130
> 2	273 (91.6)	1.32 (0.49, 3.57)
Frequency of child care attendance (days/week)
0	74 (24.8)	1.52 (0.48, 4.78)	0.058
1–2	12 (4.0)	1.50 (0.68, 3.32)
3–4	41 (13.8)	1.19 (0.62, 2.29)
≥ 5	171 (57.4)	1.29 (0.48, 3.47)
Exposed to secondhand smoke
Yes	35 (11.7)	1.47 (0.48, 4.47)	0.239
No	263 (88.3)	1.32 (0.50, 3.51)
Guardian-related variables
Mother’s working status
Working	219 (73.5)	1.28 (0.40, 3.34)	0.014
Not working	76 (25.5)	1.51 (0.52, 4.43)
Missing values	3 (1.0)
Mother’s education level
University	181 (60.7)	1.27 (0.51, 3.15)	0.004
Secondary	57 (19.1)	1.31 (0.46, 3.73)
< Secondary	57 (19.1)	1.63 (0.53, 5.04)
Missing values	3 (1.0)
Ownership status
Owner	179 (60.1)	1.30 (0.48, 3.51)	0.309
Renter	119 (39.9)	1.39 (0.51, 3.77)
Parents’ professional exposure to lead
Yes	25 (8.4)	1.41 (0.46, 4.28)	0.587
No	273 (91.6)	1.33 (0.50, 3.56)
Frequency of home cleaning
< 1/week	73 (24.5)	1.20 (0.49, 2.93)	0.041
≥ 1/week	225 (75.5)	1.38 (0.50, 3.83)
Environmental covariates
Floor dust (μg/ft^2^)
< 0.27	58 (19.5)	1.12 (0.42, 2.97)	0.027
0.27–0.54	60 (20.1)	1.34 (0.57, 3.11)
0.54–0.88	60 (20.1)	1.36 (0.51, 3.62)
0.88–1.97	60 (20.1)	1.50 (0.54, 4.21)
≥ 1.97	60 (20.1)	1.39 (0.48, 4.01)
Missing value	1 (0.1)
Windowsill dust (μg/ft^2^)
< 1.74	93 (31.1)	1.20 (0.48, 2.99)	0.002
1.74–4.48	46 (15.4)	1.18 (0.50, 2.77)
4.48–9.90	42 (14.0)	1.39 (0.46, 4.18)
9.90–25.04	37 (12.4)	1.31 (0.59, 2.88)
≥ 25.04	45 (15.1)	1.67 (0.60, 4.63)
Missing values	36 (12.0)	1.34 (0.42, 4.29)
Lead in paint
XRF < 1 mg/cm^2^	117 (39.3)	1.24 (0.45, 3.45)	0.044
XRF ≥ 1 mg/cm^2^ or paint chips < 5,000 mg/kg	139 (46.6)	1.37 (0.52, 3.61)
Paint chips ≥ 5,000 mg/kg	42 (14.1)	1.54 (0.65, 3.65)
GM, geometric mean. ^***a***^The Student *t*-test and ANOVA procedure were used to compare BPb across strata.			

Taken as a whole, the GM of WLL measured in Montreal was relatively low for fully flushed water (GM: 0.89 μg/L; 95% CI: 0.06, 12.52) as well as for stagnant water (GM: 2.21 μg/L; 95% CI: 0.14, 35.27) (Table 2). The estimated median CWLEI based on the seasonal changes of lead concentrations in fully flushed samples was 0.48 μg/kg of body weight (vs. 0.78 for stagnant samples). The estimated mean of daily water intake by children in the whole sample was 20.85 mL/kg (95% CI: 5.41, 58.44) (Table 2).

**Table 2:** Distribution of water lead level, daily water intake, and water lead intake as estimated from cross-sectional and cumulative measures of water lead exposure.

Water characteristics	*n*	p10	p25	p50	p75	p90	GM (95% CI)
Water lead level (μg/L)
Fully flushed water^*a*^	298	0.16	0.27	1.48	5.41	9.18	0.89 (0.06, 12.52)
Stagnant water^*b*^	298	0.34	0.68	2.53	7.46	12.70	2.21 (0.14, 35.27)
Daily water intake (mL/kg)	298	7.87	12.25	18.79	26.69	37.50	20.85 (5.41, 58.44)
Cumulative water lead intake (μg/kg of bw)^*c*^
Fully flushed water	298	0.07	0.12	0.48	1.37	2.78	0.44 (0.03, 6.99)
Stagnant water	298	0.16	0.27	0.78	2.06	4.09	0.77 (0.07, 8.97)
Abbreviations: GM, geometric mean; p, percentile. ^***a***^The 1-L water collected at the kitchen tap after 5 min of flushing. ^***b***^The arithmetic mean of the four consecutive 1-L samples collected after a stagnation time of 30 min. ^***c***^Estimated by taking into account the expected value of water lead concentration obtained by modeling the seasonal changes in water lead concentration over the 150 days preceding the day of the visit, and after adjusting for the presence of lead service lines (yes/no), the flow rate (continuous), the neighborhood (nominal), the type of residence (single house, row houses, multi-levels), the age of residence, the total number of people living in household, and the floor where the tap was located.							

There was a significant positive association between CWLEI and ln(BPb) in both Caucasian and non-Caucasian children, and no significant difference between the two groups (*p*-interaction = 0.57) (see Supplemental Material, Figure S3). Using the cross-sectional exposure metric (i.e., the WLL as measured at the day of visit), the fit statistic was very low (*R*^2^ = 0.03 for the crude model). This value remained low, but was slightly improved using cumulative exposure metric (*R*^2^ = 0.10 for the crude model). Both cross-sectional and cumulative measures of exposure were strongly correlated with blood lead, but the correlation coefficient, though weak, was slightly elevated when using the cumulative exposure metric (*r* = 0.31, *p* < 0.0001 and *r* = 0.26, *p* < 0.0001 for cumulative and cross-sectional metric, respectively).

CWLEI was positively associated with ln(BPb) before and after adjustment (Table 3). When modeled as a continuous variable, a 1-unit increase in CWLEI was associated with a 0.12-μg/dL increase in ln(BPb) (95% CI: 0.08, 0.17) before adjustment, and a 0.10-μg/dL increase (95% CI: 0.06, 0,14) after adjustment (Table 3). The cumulative index was estimated for 150 days preceding the day of blood collection, and therefore an increase of 1 unit in CWLEI corresponds approximately to a daily ingestion of 1/150 μg Pb/kg [i.e., 0.007 μg/kg of body weight (bw)]. Assuming the mean water consumption for a child of 21 mL/kg/day, our result could be translated into an increase of 0.10 μg/dL in ln(BPb) for each increase of 0.007/0.021 μg/L in the water lead concentration. Thus, to translate the cumulative metric of exposure (micrograms per kilogram of body weight) into the conventional unit (micrograms per liter), we could say that a 1-unit increase in water lead concentration is associated with an estimated increase of 0.30 μg/dL in ln(BPb) after adjusting for confounders. In others words, it would result in a 35% change in BPb. The categorical model indicated significant positive associations for CWLEI ≥ 0.72 μg Pb/kg body weight, with estimated increases in geometric mean BPb of 19% (95% CI: 0, 42%) and 39% (95% CI: 15, 67%), respectively, after adjustment (trend *p*-value < 0.0001) (Table 3). This cut point of 0.72 μg Pb/kg bw corresponds to 0.72/150 μg/kg/day. If the child consumes 21 mL of water/kg/day, this cut point corresponds to a water lead concentration of 0.23 μg/L [i.e., (0.72/150) × 1,000/21]. Estimated associations with WLL measured on the same day were similar to associations with the cumulative exposure metric, but model *R*^2^ values indicated a slightly better fit for the model of CWLEI versus measured WLL (*R*^2^ = 0.13 and 0.08, respectively, for the adjusted models). As a whole, the association remained unchanged when floor dust lead loading, windowsill dust lead loading, and paint-lead levels were included as covariates into the model (Table 3).

**Table 3:** Relation between blood lead concentration and both cross-sectional and cumulative metric of water lead exposure.

Water lead exposure metrics	Crude estimate (95% CI)	Adjusted estimate^*a*^ (95% CI)	Additionally adjusted for lead in paint and dust^*b*^ (95% CI)
Water lead concentration (μg/L)
Continuous^*c*^	0.07 (0.04, 0.11)	0.06 (0.02, 0.07)	0.03 (0.02, 0.06)
	*R*^2^**= 0.04	*R*^2^ = 0.10	*R*^2^ = 0.18
Quartile^*d*^
< 0.61 (reference)	1	1	1
0.61–2.31	1.07 (0.91, 1.26)	1.03 (0.86, 1.24)	0.95 (0.79, 1.14)
2.31–6.81	1.21 (1.02, 1.42)	1.20 (1.00, 1.43)	1.07 (0.89, 1.30)
≥ 6.81	1.32 (1.12, 1.56)	1.33 (1.10, 1.59)	1.23 (1.01, 1.48)
	*p*_*Trend*_ < 0.0001	*p*_*Trend*_ < 0.0001	*p*_*Trend*_ < 0.0001
	*R*^2^ = 0.03	*R*^2^ = 0.08	*R*^2^ = 0.17
CWLEI based on 80:20 ratio (μg Pb/kg of bw)
Continuous^*c*^	0.12 (0.08, 0.17)	0.10 (0.06, 0.14)	0.08 (0.03, 0.11)
	*R*^2^ = 0.10	*R*^2^ = 0.12	*R*^2^ = 0.19
Quartile^*d*^
< 0.24 (reference)	1	1	1
0.24–0.72	1.04 (0.89, 1.22)	0.95 (0.80, 1.14)	1.01 (0.85, 1.21)
0.72–1.92	1.23 (1.05, 1.45)	1.19 (1.00, 1.42)	1.09 (0.91, 1.31)
≥ 1.92	1.47 (1.25, 1.73)	1.39 (1.15, 1.67)	1.32 (1.09, 1.60)
	*p*_*Trend*_ < 0.0001	*p*_*Trend*_ < 0.0001	*p*_*Trend*_ < 0.0001
	*R*^2^ = 0.10	*R*^2^ = 0.13	*R*^2^ = 0.18
^***a***^Model of CWLEI adjusted for child’s age, child’s sex, child’s ethnicity, duration of breastfeeding, mother’s education level, frequency of daycare attendance, number of meals per day, and the season of blood collection. Model of water lead adjusted for child’s age, child’s sex, child’s ethnicity, child’s body weight, duration of breastfeeding, mother’s education level, frequency of daycare attendance, number of meals per day, and the season of blood collection. ^***b***^Additionally adjusted for floor dust loading, windowsill dust loading, and paint-lead levels. ^***c***^Estimates represent the association between water lead exposure and ln(BPb). ^***d***^Estimates are expressed as ratio of BPb.			

*Sensitivity analyses*. The relation between CWLEI and BPb was nearly similar whether 100% flushed or stagnant water was considered (see Supplemental Material, Table S1). Sensitivity analyses showed that the CWLEI markedly increased with the gastrointestinal absorption rate. However, when the gastrointestinal absorption rate was kept stable, the CWLEI was weakly influenced by changing the fraction of flushed (vs. stagnant) water ingested by children (see Supplemental Material, Figure S4). As a whole, the association between CWLEI and BPb remained stable, independent of both gastrointestinal absorption rate and fraction of flushed (vs. stagnant) water ingested by children (see Supplemental Material, Table S2). The statistics of fit (herein adjusted *R*^2^) also remained similar for the different assumptions considered (about 10–12%).

## Discussion

In this study, we used a cumulative index of exposure (CWLEI) to examine the dose–response relationship between lead concentration in the household water and children’s BPb. The geometric mean BPb of 1.3 μg/dL (95% CI: 0.5, 3.6) observed in our study is similar to the mean value reported in U.S. children 1–5 years of age between 2007 and 2010 (GM = 1.3 μg/dL; 95% CI: 1.3, 1.4) (CDC 2013). We found that CWLEI was positively associated with BPb, with a clear dose–response pattern starting at 0.7 μg Pb/kg of bw. In the range of exposure observed, for each additional increase of 1 unit in water cumulative exposure index (micrograms per kilogram of body weight), the ln(BPb) of young children is expected to increase by 0.10 μg/dL (95% CI: 0.06, 0.14) after adjustment. Thus, an increase of 1-unit in the CWLEI would result in a 10.5% increase in BPb. Based on the mean of water consumption in the whole sample (≈ 21 mL/kg/day), the model suggests that an increase of 1 μg/L in water lead concentration would result in a 35% increase in the BPb after 150 days of exposure.

We estimated that a CWLEI of 0.7–1.9 μg/kg would increase mean BPb by 19% (95% CI: 0, 42%), and a CWLEI ≥ 1.9 μg/kg would increase mean BPb by 39% (15, 67%) relative to a CWLEI < 0.2 μg/kg of bw. Given that the cumulative index was constructed over 150 days, this value of 0.7 μg/kg of bw corresponds approximately to an exposure mean of 0.005 μg/kg/day. Based on the mean daily water intake of 21 mL/kg/day, the estimated lead concentration in tap water that is expected to result in a marked association with children’s BPb after 150 days of exposure is about 0.23 μg/L for children 1–5 years of age. The sensitivity analyses showed that the direction and strength of this association were similar and consistent across different values of gastrointestinal absorption rates (50%, 75%, and 90%), and scenarios of exposure (80:20, 50:50, and 20:80).

Previous works on the association between lead concentration in drinking water and children’s BPb yielded inconsistent results (see Supplemental Material, Table S3). No previous study has considered a cumulative lead exposure index through drinking water in relation to young children’s BPb. This makes it difficult to compare our results with those of previous reports. However, the finding that a CWLEI is significantly related to BPb is not a surprise, and the positive association is consistent with most previous studies (Edwards et al. 2009; Lanphear et al. 1998, 2002; Oulhote et al. 2013). Lanphear et al. (1998) suggested that BPb are expected to increase by 1 μg/dL for each additional increase of 1 μg/L in flushed water lead, in children 12–31 months of age. However, the statistical significance of this association was borderline when the focus was only on water lead below the U.S. standard of 15 μg/L (U.S. EPA 2010). The association Lanphear et al. (1998) observed between water lead and BPb may be biased, because the BPb measured at the day of visit and taken as outcome variable was attributed to the WLL measured during the same day. However, lead concentrations in tap water are not stable and are supposed to depict summer increase, thus changing the patterns of exposure. In our previous work, we showed that this increase may reach 6 μg/L in the fully flushed water (Ngueta et al. 2014). Using the cross-sectional measure of exposure (i.e., the water lead concentration as measured on the day of the visit), our regression model indicates that each 1-μg/L increase in water lead levels multiplies the expected value of BPb by 1.06 (95% CI: 1.02, 1.07) (Table 3). In a recent study conducted in France and including children < 6 years, Oulhote et al. (2013) estimated that a 10-μg/L standard would result in a 3.5-μg/L decrease in the geometric mean BLL, and a standard of 1 μg/L would result in another 0.8-μg/L decrease. On the percentage basis, this could be very comparable to the little change that we estimated.

Other previous cross-sectional studies assessing the association between WLL and BPb in children < 6 years yielded conflicting results. Gasana et al. (2006) observed no correlation between WLL and BPb among children in inner-city communities of Miami, Florida (USA), but their study suffered from a small sample size that could mask the presence of a positive association. Their results were similar to those previously obtained by Morse et al. (1979) in Bennington, Vermont (USA). For both studies, authors did not report adjusted estimates.

In addition to the cross-sectional studies mentioned above, results from two cohort studies assessing association between WLL and PBb are conflicting. In a study conducted in Rochester, New York (USA), a strong relation between WLL and changes in BPb in children ages 6–24 months was reported (Lanphear et al. 2002). After a follow-up from 6 to 24 months of age, children who lived in housing with WLL > 5 μg/L showed BPb levels that were 1.02 μg/dL higher (20.4% of change) than those in children who had WLL ≤ 5 μg/L. In this study, the authors reported neither the water sampling procedures they used nor how they handled water variable in the statistical analysis, making direct comparison with our results more difficult. Rabinowitz et al. (1985) found no association between changes in BPb and WLL for children ages 1–24 months. However, they considered the mean of WLL measured at different occasions. Consequently, the temporal fluctuation in WLL was ignored, and the same weight was attributed to water sampled at different times.

In this study, we focused on household water, which represents an important part of children’s diet. Lead in tap water is much more bioavailable than lead in food because water is often consumed between meals or after fasting conditions (e.g., early in the morning). Data from Rabinowitz et al. (1980) suggest that adult’s fasting uptake rate can be ≥ 60% compared with rates of 10–15% in association with meals. Bruening et al. (1999) suggested that this might be similar in children. Our findings support the hypothesis that after long-term exposure, lead in tap water could result in a marked increase in children’s BPb, even if the lead concentration in tap water is very low relative to the established action levels of 10–15 μg/L (Health Canada 2013; U.S. EPA 2007; WHO 2011). This is biologically plausible given the low elimination rate of blood lead that promotes its accumulation into the bloodstream. Furthermore, the association between cumulative water lead and BPb remained unchanged after adjusting for dust lead and paint lead, suggesting that BPb is responsive to changes in drinking-water lead in this population.

The present study has several strengths that should be underlined. First, we restricted our population study to children consuming tap water exclusively. Indeed, the sole presence of lead hazard through household water is not sufficient, given that living in a house with high lead concentration in tap water does not necessary mean that the child will absorb lead. Second, we took into account the cumulative exposure over time by estimating the CWLEI, based on the modeling of seasonal changes in the marginal mean of WLL, as reported in our previous work (Ngueta et al. 2014). Although it is widely assumed that blood lead is related to recent exposure, the term “recent” may be vague, and questions remain regarding the actual period to which this expression refers. The lead absorbed several months earlier contributes to blood lead measured at a given time. As a result, a single measurement of exposure for association models has limitations. The sensitivity analysis showed that changes in either gastrointestinal absorption rates or fraction of flushed (vs. stagnant) water ingested did not influence the association between water lead and BPb, suggesting that a cumulative exposure metric may be used in other populations with different lead absorption rate and remains valid for a large range of WLL. The model *R*^2^ was 0.03 in the crude model, a value as low as the one reported in a previous study (*R*^2^ = 0.02) (Lanphear et al. 1998). No value was reported in other studies identified. Using cumulative exposure metric, we observed that the *R*^2^ was improved but still low (*R*^2^ = 0.10 in the crude model).

Ideally, serial data on BPb would be useful for assessing the influence of day-to-day changes in WLL on BPb. We did not have serial BPb data, but we used CWLEI to estimate the influence of long-term exposure to water lead on BPb instead. In addition, some other study limitations need to be considered. First, for calculation of CWLEI, we were not able to take into account the indirect water consumption (from foods and beverages); however, this might be less important for younger than for older ages. Sohn et al. (2001) estimated that water represented approximately 21%, 29%, and 31% of fluid intake in U.S. children ages 1, 2, and 3–5 years, respectively. We also used a half-life of 30 days for blood lead, knowing that this is taken from prior studies that also include adults. Despite the possibility of different lead kinetic in children < 6 years of age, we were not able to find robust data to select an alternative to 30 days for the half-life of lead in blood. Also, we focused on the lead concentration of household water for estimating the CWLEI. As a result, some exposure misclassification due to consumption of water outside the home is likely, and the direction of potential bias resulting from such misclassification cannot be predicted. Finally, our estimated regression coefficients may have been biased by unmeasured potential confounders (e.g., use of folk medicines, time spent to play outdoor) or other sources of lead exposure whose levels may have been changed during days/months preceding the day of blood collection.

## Conclusion

In 1- to 5-year-old children living in the Montreal area (Canada), we found an association between lead concentration in tap water and BPb. It was estimated that in this age group, an increase of 1 μg/L in water lead would result in an increase of 35% of BPb after 150 days of exposure. A cumulative intake of ≥ 0.72 μg Pb/kg body weight from household water—which could be achieved after daily consumption of 21 mL of water with a lead concentration ≥ 0.23 μg/L for 150 days—is associated with an increase in BPb of at least 19%. Clearly, water lead concentration well below the current drinking-water guidelines in Canada and United States could have an impact on blood lead levels of young children after long-term exposure.

## Supplemental Material

(485 KB) PDF
