# Supplementary material for: Use of a Cumulative Exposure Index to Estimate the Impact of Tap Water Lead Concentration on Blood Lead Levels in 1- to 5-Year-Old Children (Montréal, Canada)
Source: Environ Health Perspect. 2015 Jun 16;124(3):388–95. doi: 10.1289/ehp.1409144 (PMC4786982; doi:10.1289/ehp.1409144)
Supplement: (485 KB) PDF [file ehp.1409144.s001.acco.pdf]

Environ Health Perspect
DOI: 10.1289/ehp.1409144

Note to Readers: *EHP* strives to ensure that all journal content is accessible to all readers. However, some figures and Supplemental Material published in *EHP* articles may not conform to 508 standards due to the complexity of the information being presented. If you need assistance accessing journal content, please contact ehp508@niehs.nih.gov. Our staff will work with you to assess and meet your accessibility needs within 3 working days.

Supplemental Material

Use of a Cumulative Exposure Index to Estimate the Impact of Tap-Water Lead Concentration on Blood Lead Levels in 1- to 5-Year-Old Children (Montreal, Canada)

Gerard Ngueta, Belkacem Abdous, Robert Tardif, Julie St-Laurent, and Patrick Levallois

Table of Contents

Figure S1: Selection process.

Figure S2: Initial causal diagram based on available variables.

Figure S3: Scatter plot providing a crude estimation of Log(blood lead concentration) (in µg/dl) for cumulative water lead exposure index (in µg of Pb/kg of body weight) in Caucasians (blue circle) and Non-Caucasians children (red circle).

Table S1: Association between cumulative water lead exposure index and blood lead levels by assuming that children consume 100% of flushing or 100% of stagnant water.

Figure S4: Influence of changes in both gastrointestinal absorption rate and fraction of flushed (versus stagnant) water ingested on the distribution of cumulative water lead exposure index (CWLEI). CWLEI50_8020 assumes a gastrointestinal absorption rate of 50% and that children consume 80% of stagnant water and 20% of flushed water.

Table S2: Association between cumulative water lead exposure index and blood lead levels, assuming different gastrointestinal absorption rates and different fraction of flushed (versus stagnant) water ingested by children.

Table S3:

References

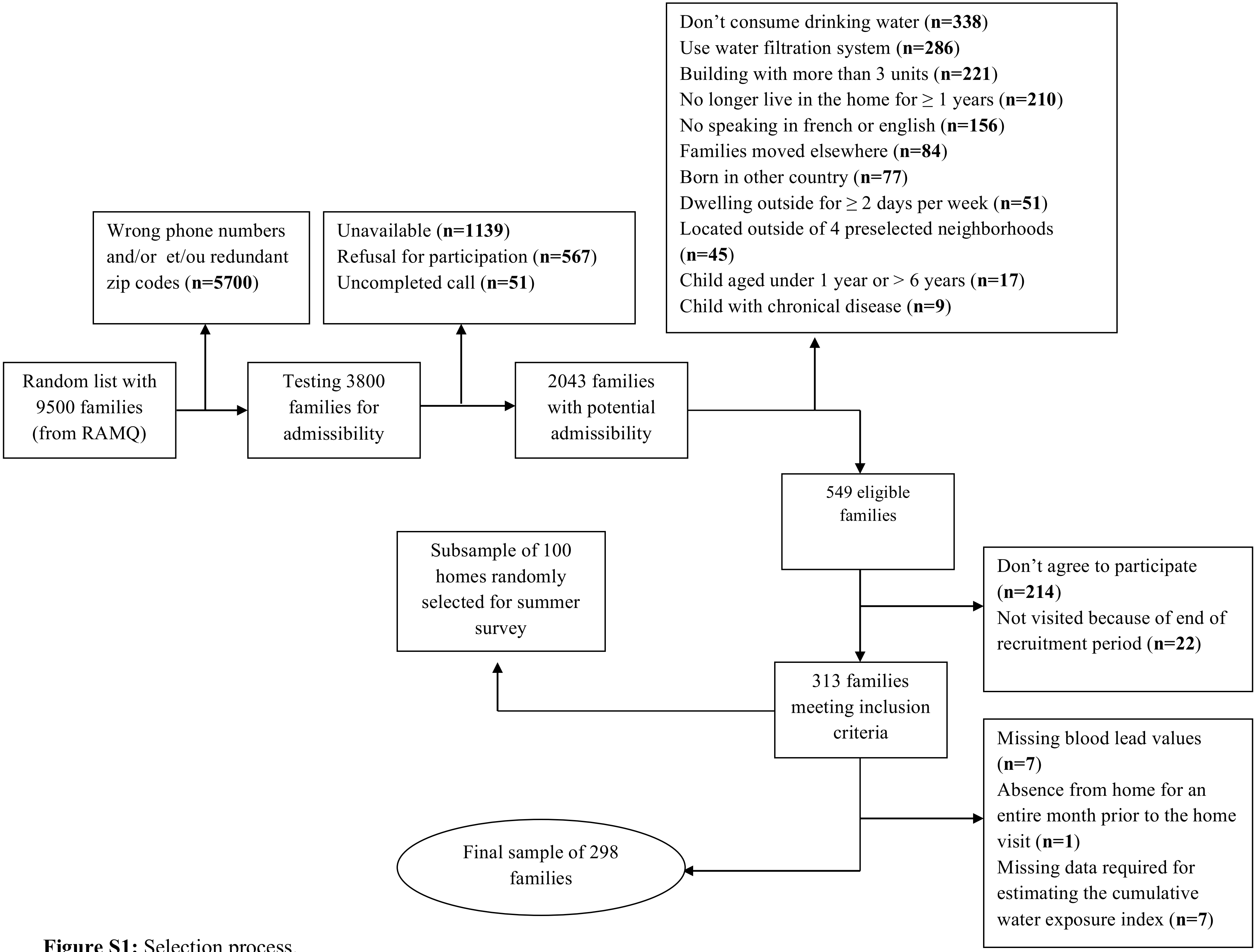

Figure S1: Selection process.

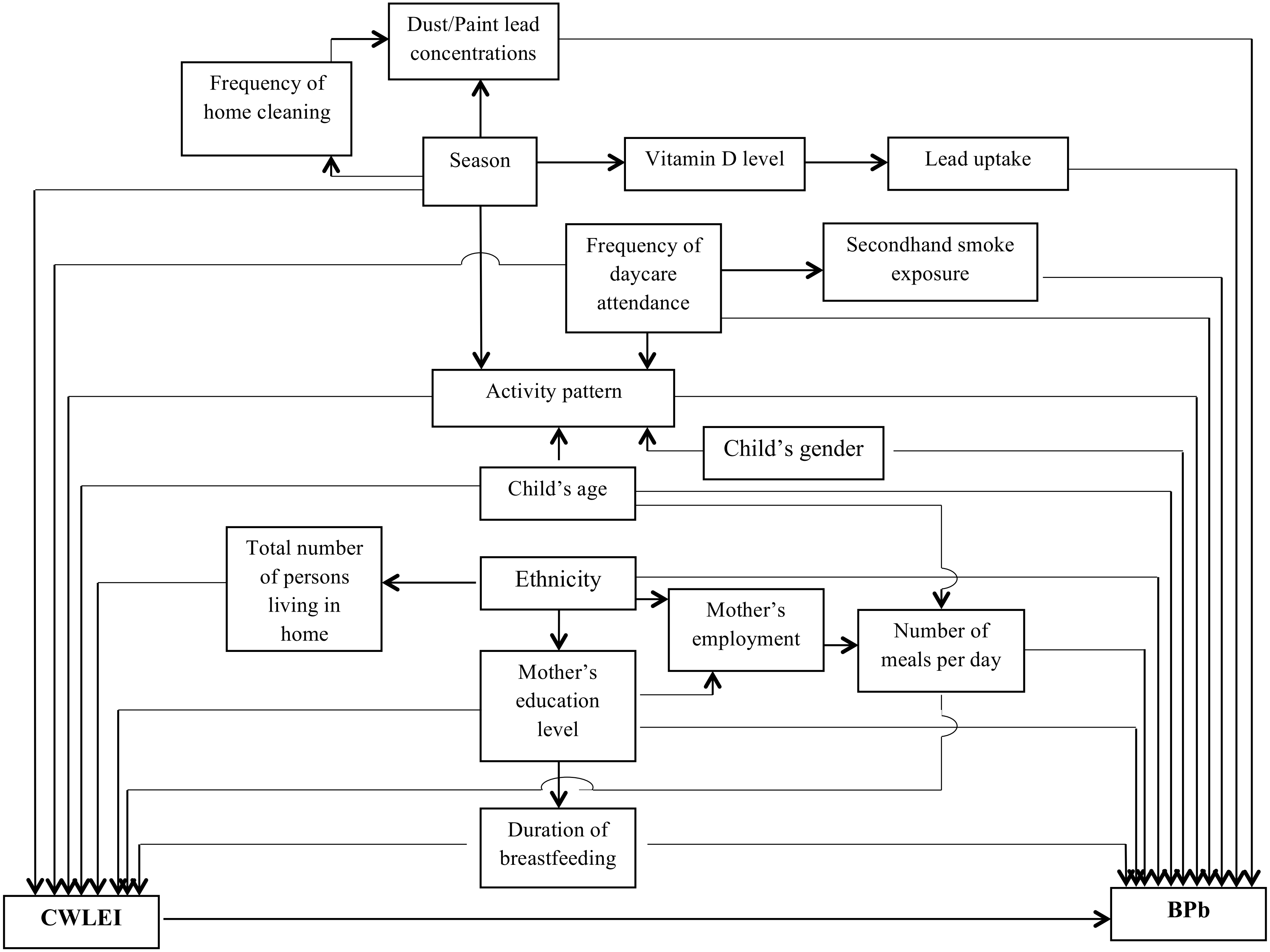

Figure S2: Initial causal diagram based on available variables.

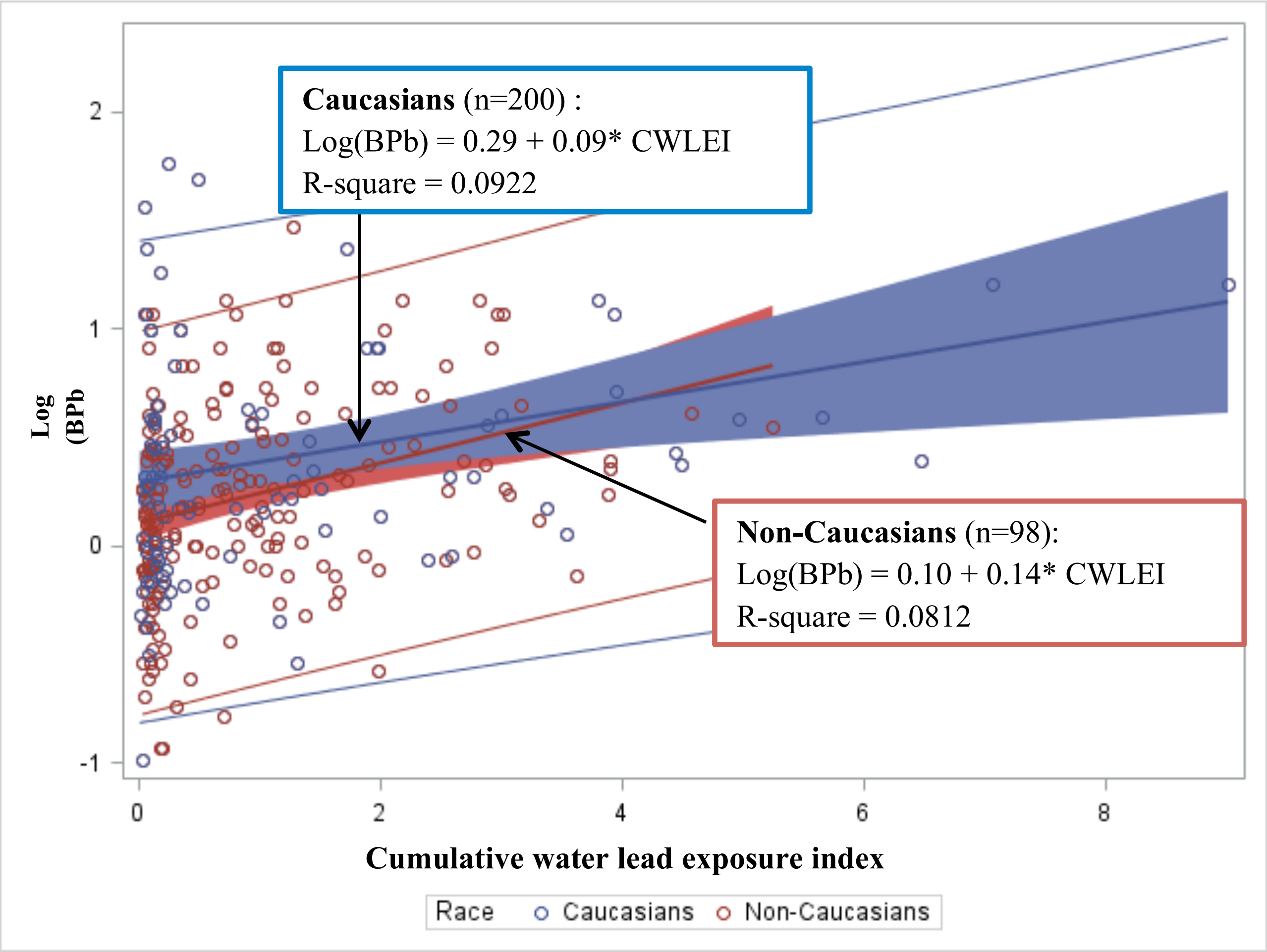

Figure S3: Scatter plot providing a crude estimation of Log(blood lead concentration) (in µg/dl) for cumulative water lead exposure index (in µg of Pb/kg of body weight) in Caucasians (blue circle) and Non-Caucasians children (red circle).

Table S1: Association between cumulative water lead exposure index and blood lead levels by assuming that children consume 100% of flushing or 100% of stagnant water.

	Cumulative water lead exposure index (in µg of Pb/kg of body weight)		Ratio of mean blood lead concentration	
	**Quartiles**	**Geometric mean within strata (95% CI)**	**Crude estimate (95% CI)**	**Adjusted estimate[c] (95% CI)**
**Flushing water[a]**				
	< 0.12 (Ref)	0.07 (0.01, 0.12)	1	1
	0.12 – 0.48	0.22 (0.12, 0.48)	1.06 (0.90, 1.24)	0.94 (0.79, 1.13)
	0.48 – 1.37	0.91 (0.47, 1.37)	1.23 (1.05, 1.45)	1.18 (0.98, 1.41)
	≥ 1.37	2.57 (1.38, 8.75)	1.49 (1.27, 1.75)	1.37 (1.13, 1.66)
			P_{Trend} <0.0001	P_{Trend} < 0.0001
			R-square = 0.1033	
**Stagnant water[b]**				
	< 0.27 (Ref)	0.16 (0.07, 0.34)	1	1
	0.27 – 0.78	0.42 (0.23, 0.78)	1.06 (0.90, 1.24)	0.94 (0.80, 1.13)
	0.78 – 2.06	1.39 (0.82, 2.37)	1.19 (1.01, 1.40)	1.17 (0.98, 1.40)
	≥ 2.06	3.81 (1.75, 8.29)	1.46 (1.24, 1.72)	1.38 (1.15, 1.66)
			P_{Trend} <0.0001	P_{Trend} < 0.0001
			R-square = 0.1031	

'Ref' stands for 'Reference group'; [a] refers to the first 1-L sampled after 5 minutes of flushing; [b]refers to the arithmetic mean of the four consecutive 1-L sampled after a stagnation time of 30 minutes; [c]Adjusted for child's age, child's gender, child's ethnicity, duration of breastfeeding, mother's education level, frequency of daycare attendance, number of meals per day and the season of blood collection.

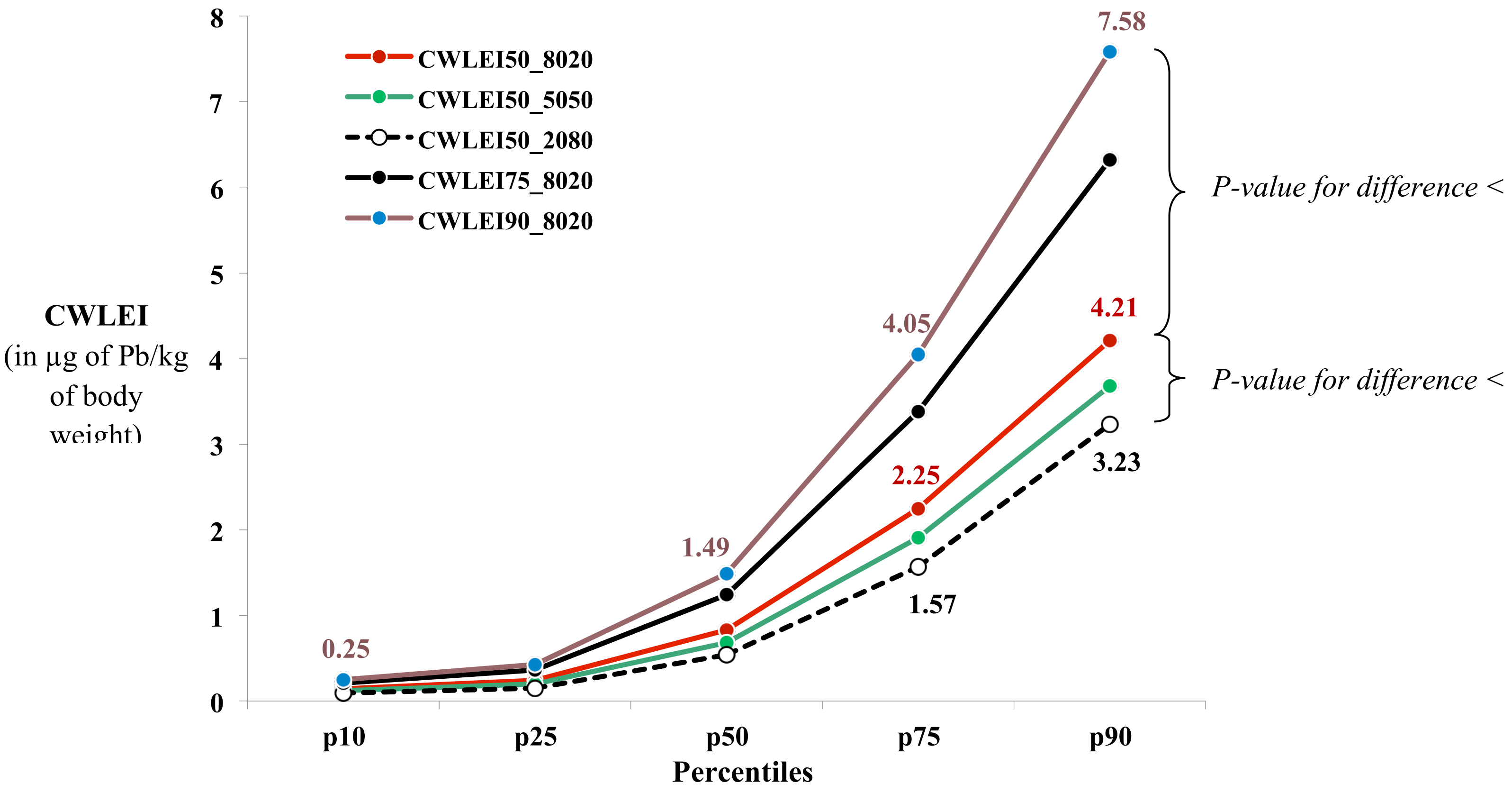

Figure S4: Influence of changes in both gastrointestinal absorption rate and fraction of flushed (versus stagnant) water ingested on the distribution of cumulative water lead exposure index (CWLEI). CWLEI50_8020 assumes a gastrointestinal absorption rate of 50% and that children consume 80% of stagnant water and 20% of flushed water.

Table S2: Association between cumulative water lead exposure index and blood lead levels, assuming different gastrointestinal absorption rates and differents fraction of flushed (versus stagnant) water ingested by children.

	**Adjusted[a] ratio of mean blood lead concentration(95% Confident intervals)**								
	Gastrointestinal absorption rate of 50%			Gastrointestinal absorption rate of 75%			Gastrointestinal absorption rate of 90%		
Pctles[b]	**80:20[c]**	**50:50**	**20:80**	**80:20**	**50:50**	**20:80**	**80:20**	**50:50**	**20:80**
< p10	1	1	1	1	1	1	1	1	1
p10 – p25	1.04(0.80, 1.34)	0.99(0.76, 1.28)	0.99(0.76, 1.30)	1.04(0.80, 1.34)	1.08(0.83, 1.42)	1.00(0.77, 1.29)	1.07(0.83, 1.39)	1.00(0.77, 1.29)	0.99(0.77, 1.29)
p25 – p50	0.96(0.76, 1.22)	0.94(0.73, 1.19)	0.94(0.74, 1.20)	0.96(0.76, 1.22)	0.98(0.76, 1.25)	0.94(0.74, 1.19)	0.98(0.77, 1.25)	0.93(0.73, 1.19)	0.94(0.73, 1.20)
p50 – p75	1.15(0.90, 1.46)	1.19(0.90, 1.52)	1.18(0.92, 1.51)	1.18(0.93, 1.50)	1.25(0.97, 1.60)	1.18(0.93, 1.49)	1.18(0.92, 1.51)	1.19(0.93, 1.51)	1.18(0.92, 1.51)
p75 – p90	1.44(1.11, 1.87)	1.26(0.93, 1.64)	1.28(0.97, 1.67)	1.39(1.07, 1.82)	1.34(1.02, 1.76)	1.27(0.98, 1.65)	1.48(1.13, 1.93)	1.26(0.97, 1.64)	1.28(0.97, 1.67)
≥ p90	1.49(1.10, 2.03)	1.48(1.08, 2.04)	1.51(1.09, 2.07)	1.49(1.10, 2.03)	1.52(1.10, 2.09)	1.51(1.11, 2.05)	1.53(1.12, 2.09)	1.48(1.08, 2.04)	1.51(1.09, 2.07)
PTrend	<0.001	<0.001	<0.001	<0.001	<0.001	<0.001	<0.001	<0.001	<0.001
R-Square	0.1218	0.1074	0.1098	0.1138	0.1075	0.1103	0.1226	0.1122	0.1100

[a]Adjusted for child's age, child's gender, child's ethnicity, duration of breastfeeding, mother's education level, frequency of daycare attendance, number of meals per day and the season of blood collection; [b]Percentiles of cumulative water lead exposure index (in µg of Pb/kg of body weight); [c]scenario assuming that children consume 80% of stagnant water and 20% of flushed water.

Table S3:

Study	Date of study	Place of study	Children's age (Sample size)	Available descriptive data for water lead concentrations	Available descriptive data for blood lead concentrations	Main result, models and fit statistics
**Cross-sectional studies**						
Levallois et al. 2014	Sept 2009 – March 2010	Montreal, QC (Canada)	1 – 5 years (n=306)	GM (95% CI): 1.60 (1.40, 1.84) µg/L	GM (95%CI) : 1.35 (1.27, 1.43) µg/dL	Statistically significant positive association between water lead and BPb<br>No fit statistics reported
Lanphear et al. 1998	August 29 – November 20, 1993	Rochester, NY (USA)	12 – 31 months (n=183)	GM : 0.0009 µg/g<br>GSD : 0.012 µg/g<br>Range : 0.0005 – 0.16 µg/g	GM: 6.2 µg/dL<br>GSD : 5.2 µg/dL<br>Range : 1.3 – 32.0 µg/dL	Positive, but borderline association (p=0.0618) between water lead and BPb : Slope = 0.0664 (SD : 0.035)<br>R^2=0.023
Oulhote et al. 2013	Sept 2008 – April 2009	France	6 – 84 months (n=484)	Range : < 1 – 74 µg/L	GM (95%CI): 14.0 (12.7, 15.0) µg/dL<br>Range : 0.26 – 30.8 µg/dL	BPb increase by 70% when lead in tap water increased from 1 to 25 g/L<br>No fit statistics reported
Gasana et al. 2006	NA	Miami, FL (USA)	< 6 years (n=75)	Mean (SD) : 4.53 (18.09) µg/L for first-draw; 1.46 (2.33) µg/L for flushed samples (30 seconds of flushing)	Mean (SD): 3.41 (1.85) µg/dL<br>Median : 3.00 µg/dL	Spearman's correlation coefficient revealed no association between water lead and BPb (rho=0.03, p=0.81 for flushed water; rho=0.005, p=0.97 for first-draw water)

Study	Date of study	Place of study	Children's age (Sample size)	Available descriptive data for water lead concentrations	Available descriptive data for blood lead concentrations	Main result, models and fit statistics
**Cross-sectional studies**						
Morse et al. 1979	May 1977	Bennington, VY (USA)	1 – 12 years (n=192)	Weighted mean : 0.07 mg/L	Range : 7 – 43 µg/dL<br>Mean : 16.1 µg/dL	No correlation between water lead and BPb :
Our study	Sept 2009 – Sept 2011	Montreal, QC (Canada)	1 – 5 years (n=298)	Flushed water :<br>GM (95%CI) : 0.89 (0.06 – 12.52) µg/L<br>Stagnant water :<br>GM (95%CI) : 2.21 (0.14 – 35.27) µg/L	GM (95% CI) : 1.34 (0.50, 3.61) µg/dL	Positive association between water lead levels and Ln(BPb) (Adjusted slope : 1.06, $p<0.0001$), $R^2=0.08$<br>Positive association between cumulative water lead index and ln(BPb) (Adjusted slope : 0.10, $p<0.0001$), $R^2=0.12$
**Follow-up studies**						
Rabinowitz et al. 1985	April 1979 – April 1981	Boston, MA (USA)	From 1 to 24 months (n=232)	Weighted mean (SD) : 5.0 (0.9) µg/L	Mean (SD) of postnatal BPb: 7.0 (5.1) µg/dL	No correlation between water lead and BPb (Spearman's correlation coefficient between 0.00 and 0.14 depending on child's age)
Lanphear et al. 2002		Rochester, NY (USA)	From 6 to 24 months (n=249)	No reported	GM (95%CI) : 2.9 (2.7, 3.1) µg/dL at baseline; 7.5 (7.0, 8.2) µg/dL at 24 months	Positive and statistically significant association between water lead and BPb ($p<0.001$)<br>No fit statistics reported

References

Gasana J, Hlaing WWM, Siegel KA, Chamorro A, Niyonsenga T. 2006. Blood Lead Levels in Children and Environmental Lead Contamination in Miami Inner City, Florida. Int J Environ Res Public Health 3(3): 228 – 234.

Lanphear BP, Burgoon DA, Rust SW, Eberly S, Galke W. 1998. Environmental exposures to lead and urban children's blood lead levels. Environ Res 76(2): 120 – 130.

Lanphear BP, Hornung R, Ho M, Howard CR, Eberle S, Knauf K. 2002. Environmental Lead Exposure during early childhood. J Pediatr 140(1): 40 – 47.

Levallois P, St-Laurent J, Gauvin D, Courteau M, Prevost M, Campagna C, et al. 2014. The impact of Drinking Water, Indoor Dust and Paint on Blood Lead Levels of Children Aged 1-5 Years in Montreal (Québec, Canada). J Expo Sci Environ Epidemiol 24(2): 185 – 191.

Morse DL, Watson WN, Housworth J, Witherell LE, Landrigan PJ. 1979. Exposure of children to lead in drinking water. Am J Public Health 69(7): 711 – 712.

Oulhote Y, Le Tertre A, Etchevers A, Le Bot B, Lucas JP, Mandin C, et al. 2013. Implications of different residential lead standards on children's blood lead levels in France: Predictions based on a national cross-sectional survey. Int J Hyg Environ Health 216 (6): 743 – 750.

Rabinowitz M, Leviton A, Needleman H, Bellinger D, Waternaux C. 1985. Environmental correlates of Infant Blood Lead Levels in Boston. Environ Research 38(1): 96 – 107.
